# Supplementary material for: Lysophosphatidic Acid Promotes the Expansion of Cancer Stem Cells via TRPC3 Channels in Triple-Negative Breast Cancer
Source: Int J Mol Sci. 2022 Feb 10;23(4):1967. doi: 10.3390/ijms23041967 (PMC8877950; doi:10.3390/ijms23041967)
Supplement: Supplementary file 1 [file ijms-23-01967-s001.zip › ijms-1542151 suppl conversion/ijms-1542151 supplementary 1.pdf]

A

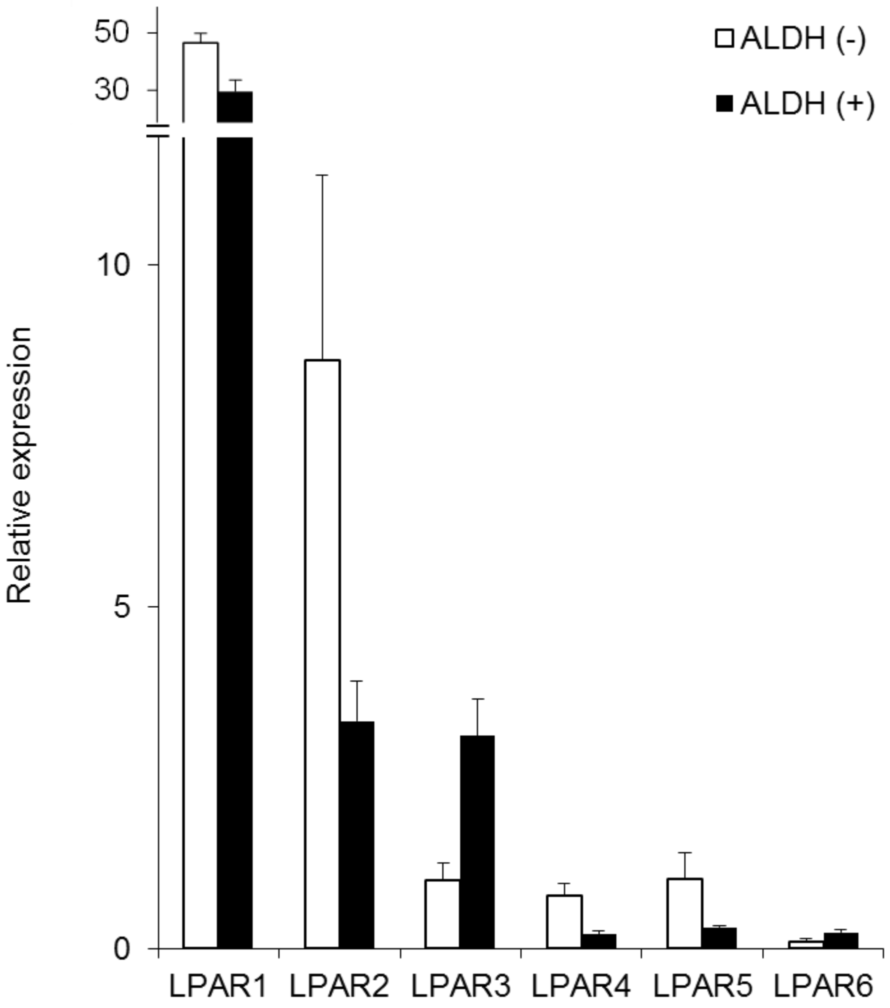

B

	ALDH (-)	ALDH (+)
LPAR1	46.6 ± 3.4	29.6 ± 4.1
LPAR2	8.6 ± 2.7	3.3 ± 0.58
LPAR3	1 ± 0.25	3.11 ± 0.53
LPAR4	0.77 ± 0.18	0.21 ± 0.05
LPAR5	1.02 ± 0.39	0.31 ± 0.03
LPAR6	0.10 ± 0.04	0.23 ± 0.05

Figure S1. LPAR expression in ALDH-negative and -positive cells of MDA-MB-231 cells. Expression level of LPAR expression showing relative to LPAR3 of ALDH-negative cells. Data represent the mean ± s.d. (n=3).

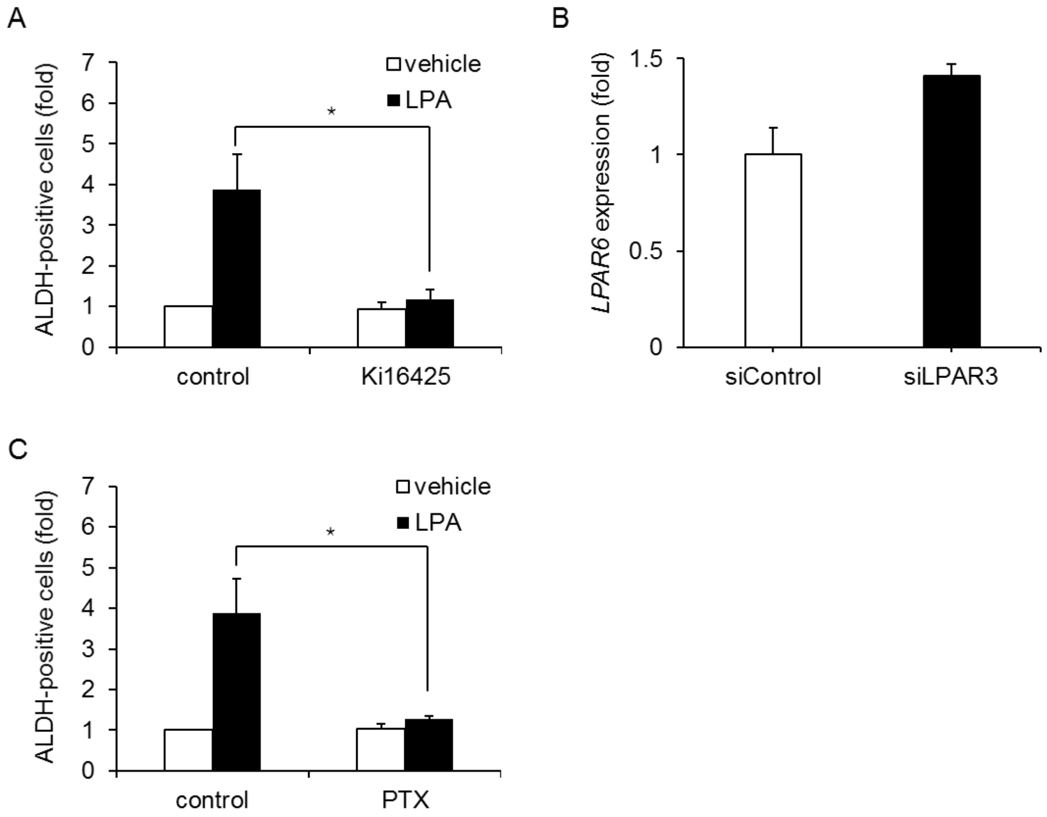

Figure S2. LPA increases ALDH-positive cells via LPAR3/Gi signaling in HCC1806 cells. (**A**) Effects of LPAR1/3 antagonists (Ki16425, 10 μM) on the number of ALDH-positive cells in HCC1806 after LPA treatment. Data represent the mean ± s.d. (n=3). (**B**) Effect of LPAR3 siRNA on LPAR6 expression. Data represent the mean ± s.d. (n=3). (**C**) Effect of PTX (100 ng/mL) on the number of ALDH-positive cells in HCC1806 cells after LPA treatment. Data represent the mean ± s.d. (n=3). $^*p < 0.05$.

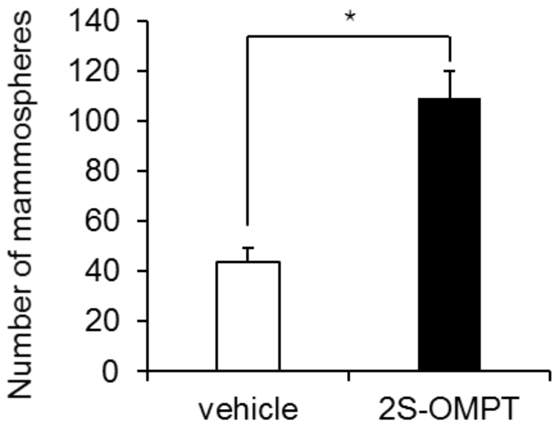

Figure S3. 2S-OMPT increases mammosphere formation in MDA-MB-231 cells. Effects of 3 μM 2S-OMPT on the mammosphere-forming efficiency of MDA-MB-231 cells. The number of mammospheres was counted using a microscope. Data represent the mean ± s.d. (n=3). *$p < 0.05$.

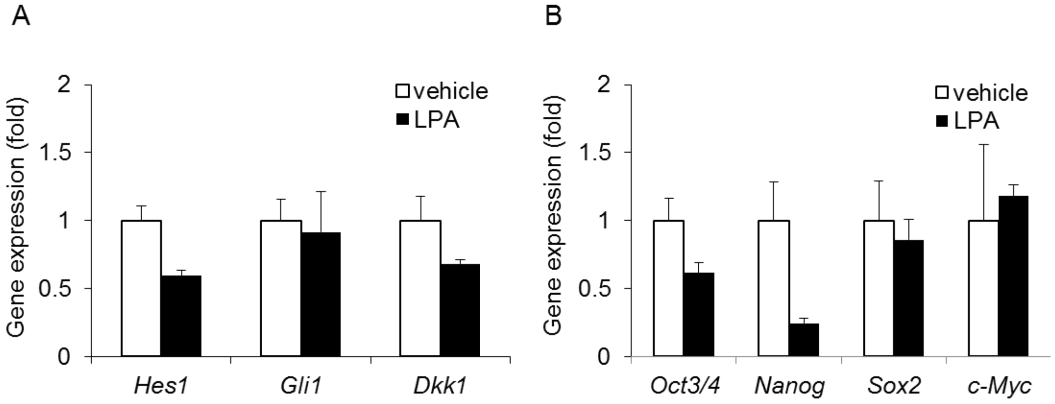

Figure S4. Effect of LPA treatment on stem cell pathway in MDA-MB-231 cells. (**A**) After stimulation with LPA (10 μM, 24 h), expression of target gene of Notch (Hes1), Hedgehog (Gli1), Wnt (Dkk1) were analyzed by qPCR. (**B**) After stimulation with LPA (10 μM, 24 h), expression of self-renewal-related transcription factors were analyzed bu qPCR. Data represent mean ± s.d. (n=3). *p < 0.05.

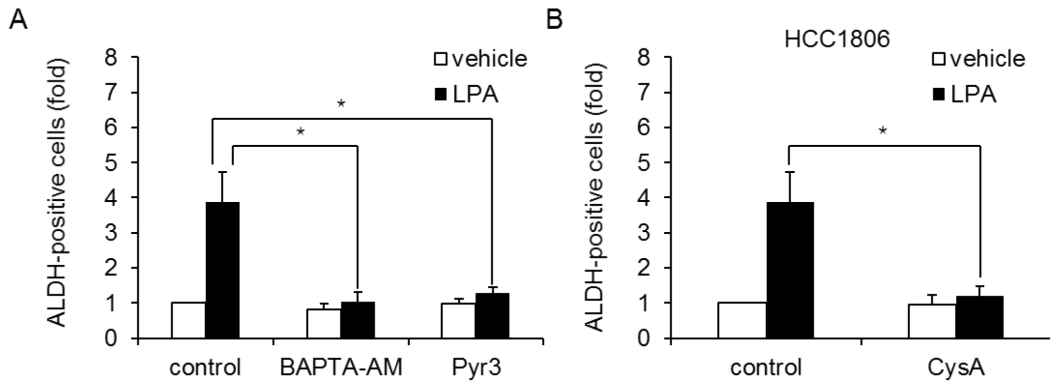

Figure S5. LPA-induced Ca^{2+}/calcineurin/NFAT signaling in HCC1806 cells. (**A**) Effects of BAPTA-AM (1 μM) and Pyr3 (1 μM) on the LPA-induced increase in ALDH-positive cells. Data represent the mean ± s.d. (n=3). (**B**) Effect of CysA (10 μM) on LPA-induced increases in ALDH-positive cells. Data represent the mean ± s.d. (n=3). *$p < 0.05$.

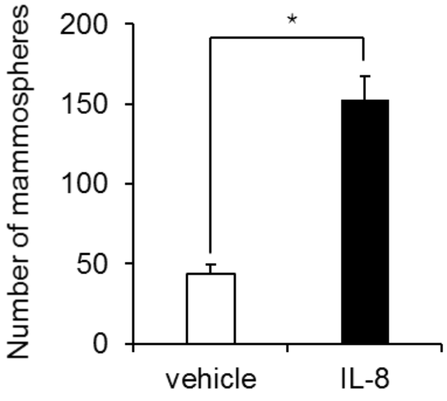

Figure S6. IL-8 increases mammosphere formation in MDA-MB-231 cells. Effects of 100 ng/ml IL-8 on the mammosphere-forming efficiency of MDA-MB-231 cells. The number of mammospheres was counted using a microscope. Data represent the mean ± s.d. (n=3). *$p < 0.05$.

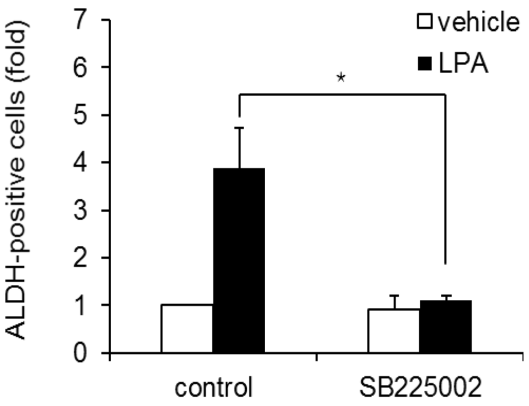

Figure S7. Role of IL-8 on LPA-induced increases in ALDH-positive cells in HCC1806 cells. Effect of the IL-8 receptor antagonist SB225002 (1 μM) on the LPA-induced proportion of ALDH-positive cells in HCC1806 cells. Data represent the mean ± s.d. (n=3). *$p < 0.05$.

Supplementary Table S2: Primer sequences used for qPCR analysis.

	forward	reverse
LPAR1	GCTGGTGATGGGACTTGGAAT	CAACCCAGCAAAGAAGTCTGC
LPAR2	GCATGGCAGAGCATGTCA	TGCAGGACTCACAGCCTAAA
LPAR3	AGGACACCCATGAAGCTAATGAA	GCCGTCGAGGAGCAGAAC
LPAR4	CCAGACACGTTTGGAGAAGC	TCTGGATCCTAGTCCTCAGTGG
LPAR5	CGCCATCTTCCAGATGAAC	TAGCGGTCCACGTTGATG
LPAR6	TCTGGCAATTGTCTACCCATT	TCAAAGCAGGCTTCTGAGG
ATX	CTTTCGGCCCTGAGGAGAGTA	AGCAACTGGTCTTTCCTGTCT
LPP1	CAAGACTCTTACGCCCCACACT	GAGAAAGGCCCACATAAATGGA
LPP2	CCGAGGCCAGGTGTCTTT	ACACCATGCAGTACATCCCAAA
LPP3	TGGTGGCCTGCTGCATAGT	GGAGAGCGTCGTCTTAGTCTTGA
Hes1	AGCGGGCGCAGATGAC	CGTTCATGCACTCGCTGAA
Gli1	GTGCAAGTCAAGCCAGAACA	ATAGGGGCCTGACTGGAGAT
Dkk1	GGGCGGGAATAAGTACCAG	CATAGCGTGACGCATGCAG
Oct3/4	ACATCAAAGCTCTGCAGAAAGAA	CTGAATACCTTCCCAAATAGAACCC
Nanog	CAGAAGGCCTCAGCACCTAC	ATTGTTCCAGGTCTGGTTGC
Sox2	AACCCCAAGATGCACAACTC	GCTTAGCCTCGTCGATGAAC
c-Myc	CACGAAACTTTGCCCATAGC	GCAAGGAGAGCCTTTCAGAG
TRPC3	CAAGAATGACTATCGGAAGC	GCCACAAACTTTTTTGACTTC
IL-8	TTGGCAGCCTTCCTGATTTC	AACTTCTCCACAACCCTCTG
CXCR2	AGGTCAGAAGTTTCATCGTCAAG	AAAGCTGTCACTCTCCATGTTAA
